# Characterization of diffusivity and mechanical properties of polyethylene glycol hydrogel conformal coatings over time for application in beta cell replacement therapy for type 1 diabetes

**DOI:** 10.1007/s12195-025-00878-7

**Published:** 2025-11-16

**Authors:** Noa H. deHaseth, Grisell C. Gonzalez, Aaron A. Stock, Ana L. Claure, Gabriela Orlando, Alice A. Tomei, Noel M. Ziebarth

**Affiliations:** 1 Department of Biomedical Engineering, University of Miami College of Engineering, Miami, FL USA; 2 Diabetes Research Institute, University of Miami Miller School of Medicine, Miami, FL 33136 USA; 3 Department of Surgery, University of Miami Miller School of Medicine, Miami, FL 33136 USA; 4 Department of Microbiology and Immunology, University of Miami Miller School of Medicine, Miami, FL 33136 USA; 5 Department of Ophthalmology, University of Miami Miller School of Medicine, Miami, FL 33136 USA

**Keywords:** Atomic force microscopy, Fluorescence recovery after photobleaching, Young’s modulus of elasticity, Diffusion, Microencapsulation, Insulin-secreting cell

## Abstract

**Purpose:**

Type 1 diabetes (T1D) is an autoimmune disorder that causes selected destruction of insulin-secreting pancreatic beta cells leading to insulin deficiency, hyperglycemia, and long-term complications. T1D has no cure and is primarily self-managed with blood sugar monitoring and exogenous insulin injections, which do not enable proper metabolic control and decreases patient’s and caregivers’ quality of life. Beta cell replacement through islet transplantation could cure T1D if current limitations such as the need for chronic systemic immunosuppression to prevent rejection and recurrence of autoimmunity are addressed. A potential new treatment addressing these limitations is based on transplantation of donor islets encapsulated in hydrogels with suitable and stable permselectivity and mechanical properties. Specifically, these hydrogel coatings must be (*1*) permeable to nutrients, insulin and glucose, necessary for coated cell viability and functionality, but impermeable to antibodies, to enable immune isolation, and (*2*) resistant to degradation, over time.

**Methods:**

This study uses Fluorescence Recovery after Photobleaching (FRAP) and Atomic Force Microscopy (AFM) to determine the diffusion coefficient and Young’s modulus of elasticity of individual model beads and primary and pseudoislets conformally coated with polyethylene glycol (PEG) over an extended period of time to evaluate the stability and viability of this novel therapeutic method for beta cell replacement without immunosuppression in T1D.

**Results:**

The conformal hydrogel coatings remained functional and did not deteriorate over the 100-day time period, showing a promising stability to enable long-term immunoisolation of encapsulated islets.

**Conclusions:**

This report demonstrated a novel measurement technique capable of assessing the mechanical and transport properties of individually coated samples, giving a more precise characterization of inherent variabilities within a sample population. Moreover, the approach is adaptable to other therapeutic cell clusters and organoids, supporting broader applications in cell transplantation therapies and offering a robust method for batch release validation in clinical applications.

## Introduction

Type 1 diabetes (T1D) is an autoimmune disorder that targets the insulin-producing β cells found in the pancreatic islets of Langerhans in a patient’s body, leading to loss of endogenous insulin secretion and glucose homeostasis [1]. Currently, the exact cause of T1D is unknown, and there is no cure. There is evidence of genetic predispositions for developing T1D, and T1D is the most prevalent form of diabetes in children and young adults [2]. To treat T1D, exogenous insulin replacement therapy is prescribed along with daily blood sugar and carbohydrate intake monitoring. T1D is primarily self-managed, and as a result, it requires the patient and caregivers to persistently meet the demands of T1D treatment, which can affect them mentally, physically, and socially [3, 4]. Today, patients with T1D are at increased risk of chronic kidney disease, heart complications, limb amputation, eye complications, and death [2].

Transplanting allogeneic pancreatic islet cells isolated from cadaveric pancreata to replace the lost insulin-secreting β cells improves metabolic control and quality of life [5, 6]. Islet transplantation is an already established T1D treatment reserved for a small percentage of patients with brittle T1D that cannot meet the targeted metabolic control with insulin therapy and glucose monitoring. Clinically, primary human islets or stem cell-derived islets are implanted through the portal vein [7, 8]. This is a relatively safe and minimally invasive procedure that could be a potential cure for T1D. However, the procedure requires the use of lifelong immunosuppression therapy to prevent islet allorejection and recurrence of autoimmunity, leaving the patient vulnerable to illness and organ toxicity [5, 9–11].

Islet encapsulation may reduce or even eliminate the complications of the current clinical islet cell transplantation procedure. Encasing the islet cell in an inert, biocompatible, non-immunogenic material would act as a physical barrier against the patient’s immune system [12, 13]. This biomaterial coating must act as a semi-permeable membrane that allows insulin, glucose, oxygen, nutrients, and waste products to pass through while preventing diffusion of antibodies and ingress of immune cells [14, 15]. Islet encapsulation would effectively eliminate the need for chronic systemic immunosuppression therapy by inhibiting host immune rejection of the allogeneic islet transplants. However, managing islet hypoxia and shear stress, coating biomaterial-mediated inflammation and foreign-body-triggered fibrosis, while preventing coating biomaterial degradation for the long time periods needed to promote long-term efficacy of transplanted encapsulated islets, are critical for the successful implementation of islet encapsulation [16]. Preventing coating degradation is of outmost importance so that coatings can preserve their physical barrier function while maintaining their selective permeability for long-term protection from the host’s immune system and functionality.

While several different encapsulation materials and strategies have been reported, overall, evaluation of their long-term outcomes is poorly investigated, especially in large animals and in humans. Common islet encapsulation materials include natural and synthetic polymers and hydrogels, such as alginate, chitosan, and polyethylene glycol (PEG) [14, 17–20]. Agarose, collagen, and gelatin are also being explored. Strategies for encapsulation that have been explored are categorized based on the scale level of encapsulation, with nano-encapsulation involving nanoscale coatings around single islet cell, micro-encapsulation involving micron-size coatings, and macroencapsulation involving the encapsulation of several islet cells [16]. The reported longevity of the transplanted biomaterial coatings differs depending on the specific formulation and transplant site [12]. Polyethylene glycol (PEG) is considered the gold standard in synthetic biomaterials due to its resistance to protein absorption, adjustable synthesis, and cytocompatibility and can be employed for islet encapsulation as non-degradable PEG hydrogels. However, despite the non-degradable design of PEG hydrogels for encapsulation, it was reported that these PEG gels can degrade in certain situations, such as in presence of reactive oxygen species (ROS) [21]. Tomei *et al* have developed a PEG hydrogel microencapsulation technique that generates coatings on islets and other cell clusters that conform to the cluster shape and size, a technique known as conformal coating. This platform technology generates a 10-50µm thick capsule around the islet, at least one order of magnitude thinner than other microencapsulation technologies, to optimize nutrient exchange and minimize encapsulated islet graft volumes for transplantation and showing great promise as a novel T1D therapeutic in several preclinical islet transplantation models [14, 22, 23]. However, the long-term stability of these conformal coatings across different fabrication batches necessary for stable immunoprotection has not been examined before.

This study aimed to (*1*) investigate the diffusivity and mechanical properties of the conformal hydrogel coating on single islet model polymeric beads over time *in vitro*, (*2*) quantify the batch-to-batch variability of these properties and if this also changes over time, and (3) validate the conformal coating for use in the encapsulation of pseudoislets and primary pancreatic islets. To accomplish these aims, we measured the Young’s modulus of Elasticity (YM), the diffusion coefficient (D), and the permeability of the conformal capsules to relevant molecules (glucose and insulin) over varying time points up to 100 days after capsule fabrication and across different encapsulation production batches .

## Materials and Methods

### Study Design

To evaluate the long-term stability, or longevity, of the conformal coating, we evaluated the change in the YM, D, and P using encapsulated polystyrene beads ranging in diameter from 50-200 μm (Thermo Fisher Scientific, Waltham, MA). These beads are chemically inert and provide a rigid substrate necessary for YM measurements of the conformal coating. The beads were prepared using a mix of various sizes of polystyrene beads to mimic the distribution of human islet sizes, detailed in previous literature [26–28]. Bead sizes (μm): 50 μm, 100 μm, 150 μm, and 200 μm. 3%, 20%, 37%, and 40% were added respectively to 5% BSA solution. The beads were coated with PEG hydrogels (as described in Section "Conformal Coating Emulsion Method") and then incubated in cell culture media.

Conformal coated beads were evaluated at specific time points of incubation: 2 days, 10 days (10-11 days), and 100 days (98-119 days), as indicated in Table 1. These time points are deliberate as they are important time points for islet transplantation. For long-term animal studies using encapsulated islet cells, transplantation would occur 2 days after harvesting, encapsulating, and incubating. Graft functionality is usually evaluated for up to 100 days after transplantation.

**Table 1:** Time points and sample sizes (number of conformal coated beads) for the Young’s modulus of Elasticity (YM), diffusion coefficient (D), and partition coefficient (P) measurements performed

Incubation period (days) Median (range)	Sample size
2	YM: 40 D: 108 P: 102
10 (9-11)	YM: 34
100 (98-119)	YM: 58 D: 61 P: 59

One day prior to measurement, the samples were immobilized (explained in more detail in Section "Immobilization and Plating") in agarose gel (ThermoFisher Scientific, Inc) on a 35mm tissue culture dish (ThermoFisher Scientific, Inc) and placed back into the incubator at 37C and 5% CO_2_. On the day of the measurements, Young’s modulus of Elasticity (YM), diffusion coefficient (D) in FITC-insulin and NBD-glucose, and partition coefficient (P) were quantified using the protocols described in Sections "Atomic Force Microscopy" and "Fluorescence Recovery After Photobleaching", respectively.

Showing that these variables remain constant over time demonstrates that the PEG hydrogel coating is stable and viable for long-term immunoprotection. Showing that these variables are consistent between batches, validates the encapsulation production process.

Pseudo and primary islets were also tested to validate approach feasibility on live samples relevant for therapeutic application. NIT-1 were cultured in F-12K Medium (ATCC), 2 mM L-glutamine, 10% FBS, 100 units/ml penicillin, and 100 µg/ml of streptomycin (ThermoFisher, Waltham, MA, USA). Human islets and media were purchased from Prodo Laboratories Inc. (Aliso Viejo, CA). The 500mL bottle of Prodo Islet Media (Standard) (PIM(S)) media was supplemented with 25mL of PIM(ABS), 5mL of PIM(G) and 6ml of PIM(3X). Encapsulated cell clusters were immobilized and conformally coated in the same manner as the beads. NIT-1 insulinoma pseudoislets generated as previously reported were conformal coated and also used to evaluate YM (*n* = 16), D (*n* = 16), and P (*n* = 16) after two days of culture at 37C and 5% CO_2_ [29]. Additionally, human primary islets obtained from Prodo labs were incubated for 2 days after isolation, conformally coated, immobilized, and then measured to determine YM (*n* = 11).

### Conformal Coating Emulsion Method

This method has been described in detail previously [23]. Briefly, 8-arm 10kDa PEG-maleimide functionalized at 75% (JenKem; Plano, TX) was crosslinked at 15% with PEG-dithiol 2kDa (Jenkem; Plano, TX). Model polystyrene beads or cell clusters were suspended in this minimally crosslinked PEG solution and passed through the conformal coating device (Biorep, custom made) with external coaxial flow of 10% Span 80 in polypropylene glycol (PPG) [23] while a gelling emulsion composed of dithiothreitol (DTT) in 10% Span 80 in PPG was dispensed coaxially to promote complete PEG gelation. The now coated samples were collected in a beaker and incubated for 12 minutes. The samples were then purified by stirring in mineral oil (Millipore Sigma; St. Louis, MO) and HBSS − / − , and by centrifugation, and finally washed with HBSS-/- before culturing in cell culture medium on a T75 culture flask (ThermoFisher Scientific, Inc) at 37C and 5% CO_2_. Images of beads before and after the coating process can be found in Appendix Fig. 6.

### Immobilization and Plating

Tissue culture dishes (35 mm diameter) were placed on a 25°C hot plate. 50 μL of 0.5% agarose solution was deposited onto the dish and spread evenly using a cell scraper, approximating a gel depth of 0.05mm. The conformal coated sample was taken out of the incubator and transferred from the cell culture flask to an Eppendorf tube (1.5ml) to remove culture media supernatant by centrifugation. A 20µL pipette fit with a 250µL tip was used to transfer the sample to the dish, dispersing the sample evenly in different areas throughout the dish. The hot plate was turned off and the dishes were left to cool for 30 minutes, after which the dishes were placed at 4C for 30 minutes to enable agarose gelation and sample immobilization. For YM measurements, this process was performed an hour before the experiment. For D and P measurements, this process was performed the day before the experiment. After incubating at 4°C for 30 minutes, the analyte, FITC-insulin (i3661, Sigma-Aldrich, St. Louis, MO), or NBD-glucose (186689-07-6, Abcam Ltd, Cambridge, UK)), at a concentration of 1mg/mL, was carefully pipetted into the dish with immobilized samples to enable analyte diffusion until equilibrium was reached to measure D and P in the coating hydrogels. The dish was then wrapped in foil and placed in the incubator until measurements were made.

### Atomic Force Microscopy

**Fig. 1:**
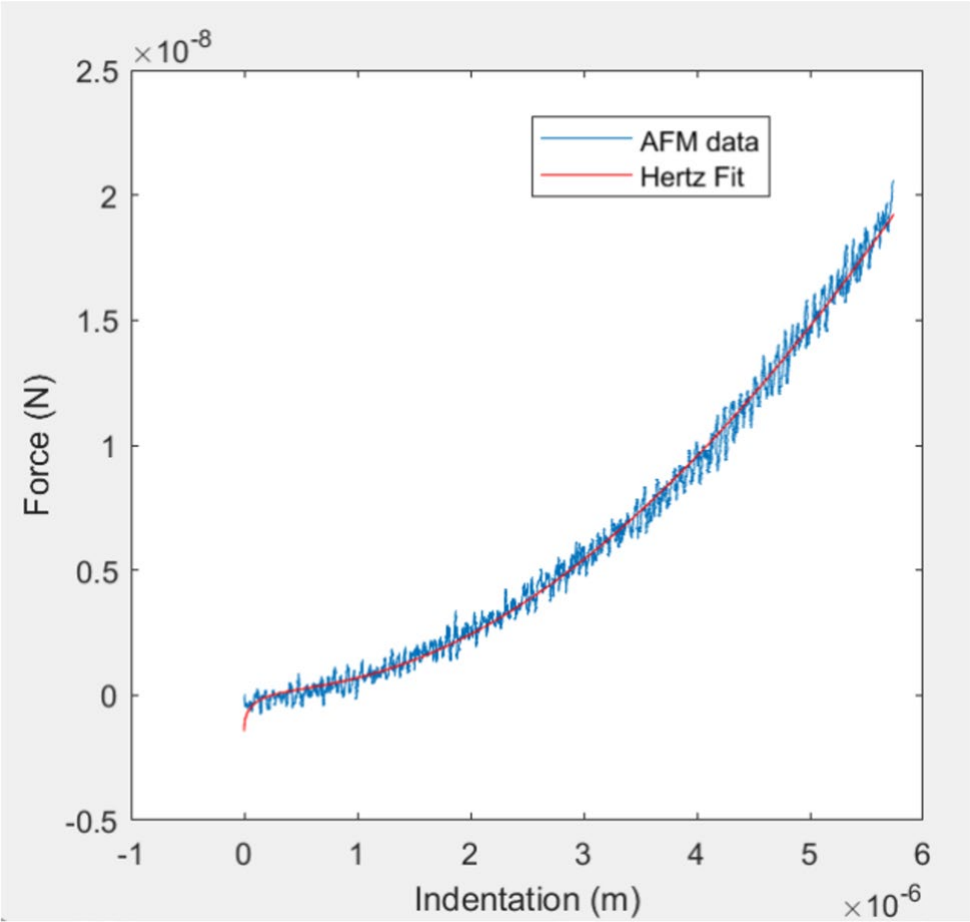
AFM force indentation curve and resulting Hertz model fit

Atomic force microscopy (AFM) was used to characterize the Young’s Modulus of individual coated beads or cell clusters samples across different time points. Young’s Modulus is a characteristic of biomechanical behavior and a measure of the stiffness of a material. It is defined as the proportion of stress over strain and gives insight into the material density and structure [24, 25]. A custom-fabricated AFM instrument optimized for biomedical applications was used, as previously described [30]. The samples were indented using a spherical indenter of 2 μm diameter (k = 0.1N/m, Novascan Technologies, Inc., USA) with a piezoelectric actuator (60μm maximal expansion, P-841.40, Physik Instrumente, Germany) at an approach and retract speed of 15 μm/s and a maximum photodiode voltage of 1V, corresponding to a force of approximately 12nN and an indentation range of 0.1-1µm, depending on sample stiffness. As this is an indentation-based characterization method, model polystyrene beads acted as the rigid substrate underneath the conformal coating, giving reliable measurements. The plated sample was fitted into the AFM system and three areas were measured on each, individual conformal coated bead. Each area was indented 15 times with the resulting values recorded. Using custom MatLab software, the resulting approach data output was converted to force-indentation curves and fit to the Hertz model for a spherical indenter (Fig. 1), shown below:

$$F=\frac{4E\sqrt{R}}{3\left(1-{v}^{2}\right)}{D}^{3/2}$$

where F is the force in Newtons, v is the Poisson’s ratio (0.49), D is the indentation (m), R is the radius of the indenter (m), and E is the Young’s modulus of elasticity (Pa). Outliers were omitted using the interquartile range (IQR) method: any value 1.5 times above the third quartile or 1.5 times below the first quartile are considered outliers [31, 32].

### Fluorescence Recovery After Photobleaching

**Fig. 2:**
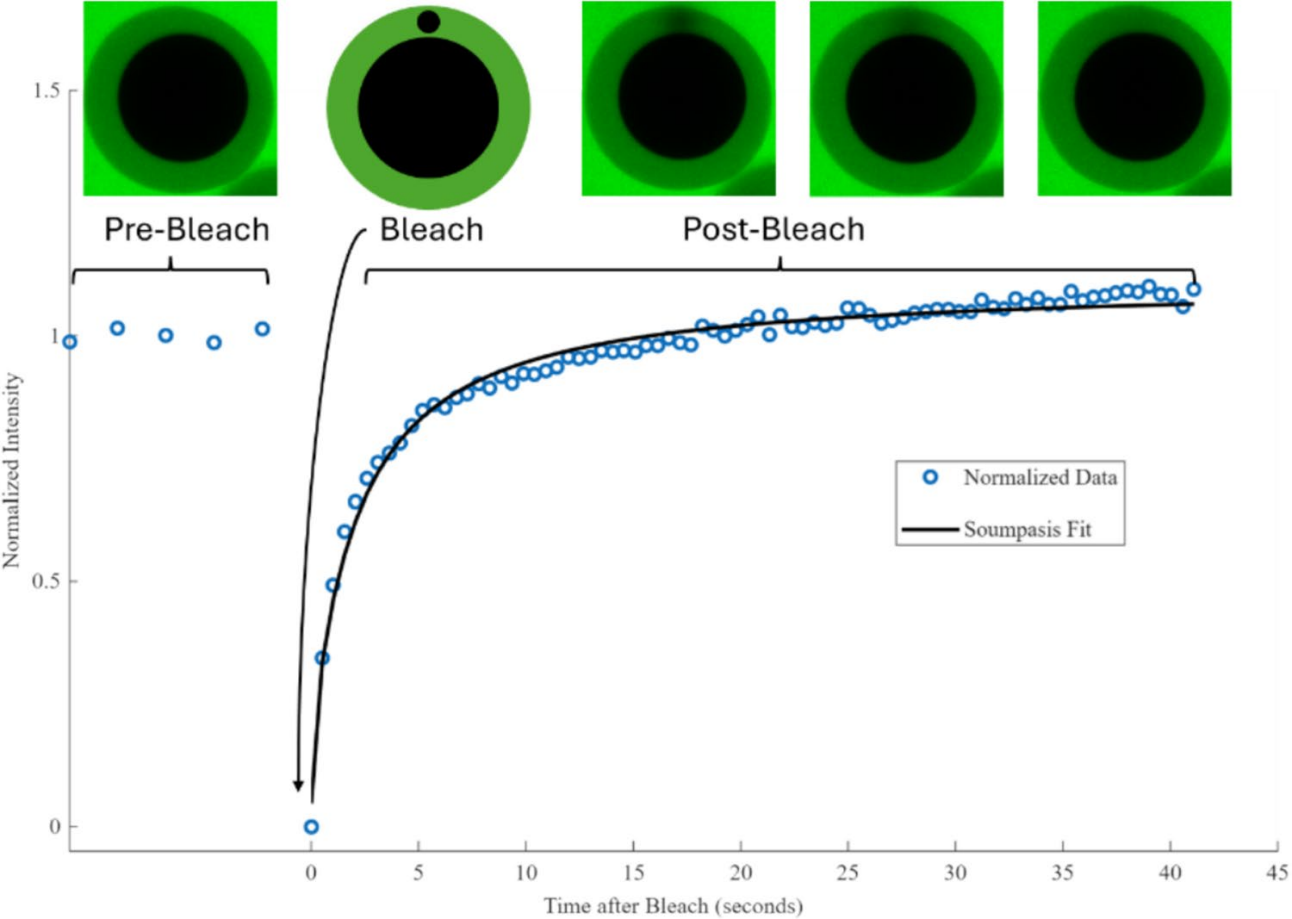
FRAP data and resulting Soumpasis Model Fit. Representative images of what the encapsulated bead looks like in each stage of measurement are highlighted above the graph

Fluorescence recovery after photobleaching (FRAP) was used to determine the diffusion coefficient (D) of the conformal coatings of individual beads or cell clusters across the different time points of insulin and glucose. The diffusion coefficient is a measure of how efficiently a substance can diffuse through a given unit of area per unit time. FRAP was performed using a Leica SP5 confocal (Leica Microsystems; Wetzlar, Germany). The central plane of each sample was found with a z-stack. A circular region of interest (ROI) of 10µm diameter was then placed in the capsule and bleached. The resulting intensity over time data was recorded (Fig. 2).

Three locations were measured per capsule. A custom MatLab code using the Soumpasis method was used to calculate the diffusion coefficient, defined below [33–35]:

$$\text{f}\left(\text{t}\right)={\text{e}}^{-\frac{2{\uptau }_{\text{D}}}{\text{t}}}\left[{\text{I}}_{0}\left(\frac{2{\uptau }_{\text{D}}}{\text{t}}\right)+{\text{I}}_{1}\left(\frac{2{\uptau }_{\text{D}}}{\text{t}}\right)\right]$$

where τ_D_ is the characteristic diffusion time and I_0_ and I_1_ are the modified Bessel functions. The diffusion coefficient (D) is calculated with:

$$\text{D}=\frac{{\text{w}}^{2}}{4{\uptau }_{\text{D}}}$$

where w is the diameter of the ROI.

The partition coefficient (P) in this study, gives a measure of how freely a molecule is able to move in and out of the conformal coatings. The partition coefficient (P), the ratio of intensity between the inside of the capsule and the outside of the capsule, was also calculated, as follows:

$$P=\frac{{I}_{capsule}}{{I}_{outside}}$$

The permeability of the membrane is directly proportional to the diffusion coefficient and partition coefficient[36]. For diffusivity and partitioning studies, glucose and insulin were selected as they are critical molecules for conformal coatings to enable glucose-stimulated insulin secretion of encapsulated islets as valuable T1D treatment. This method was used to measure samples that had been incubated for either 2 or 100 days.

### Statistical Analysis

Output data was fit to the Hertz and Soumpasis equations for AFM and FRAP, respectively. Upon passing a visual inspection of the resulting curve fits, data was included in statistical analysis. Outlier analysis was performed on the measurements of each of the three areas per encapsulated bead obtained via AFM. To represent the value of each encapsulated bead, the average of these measurements was calculated after excluding outliers. Additionally, outlier analysis was applied to the batch data for both AFM and FRAP measurements. Outliers were identified as values falling below the first quartile minus 1.5 times the interquartile range or above the third quartile plus 1.5 times the interquartile range [31, 32]. Statistical hypothesis analysis was performed in RStudio using the Student’s t-test, two-way ANOVA, or Kruskal-Wallis test, along with Tukey or Games-Howell post-hoc analysis, as appropriate. The null hypothesis tested assumed that there was no difference in means between groups. Significance was determined with a threshold of p < 0.05, indicated using a single asterisk. Additionally, a double asterisk indicates a p-value < 0.01, a triple asterisk indicates a p-value < 0.001, and a quadruple asterisk indicates a p-value < 0.0001.

## Results

### Stability Over Time

**Fig. 3:**
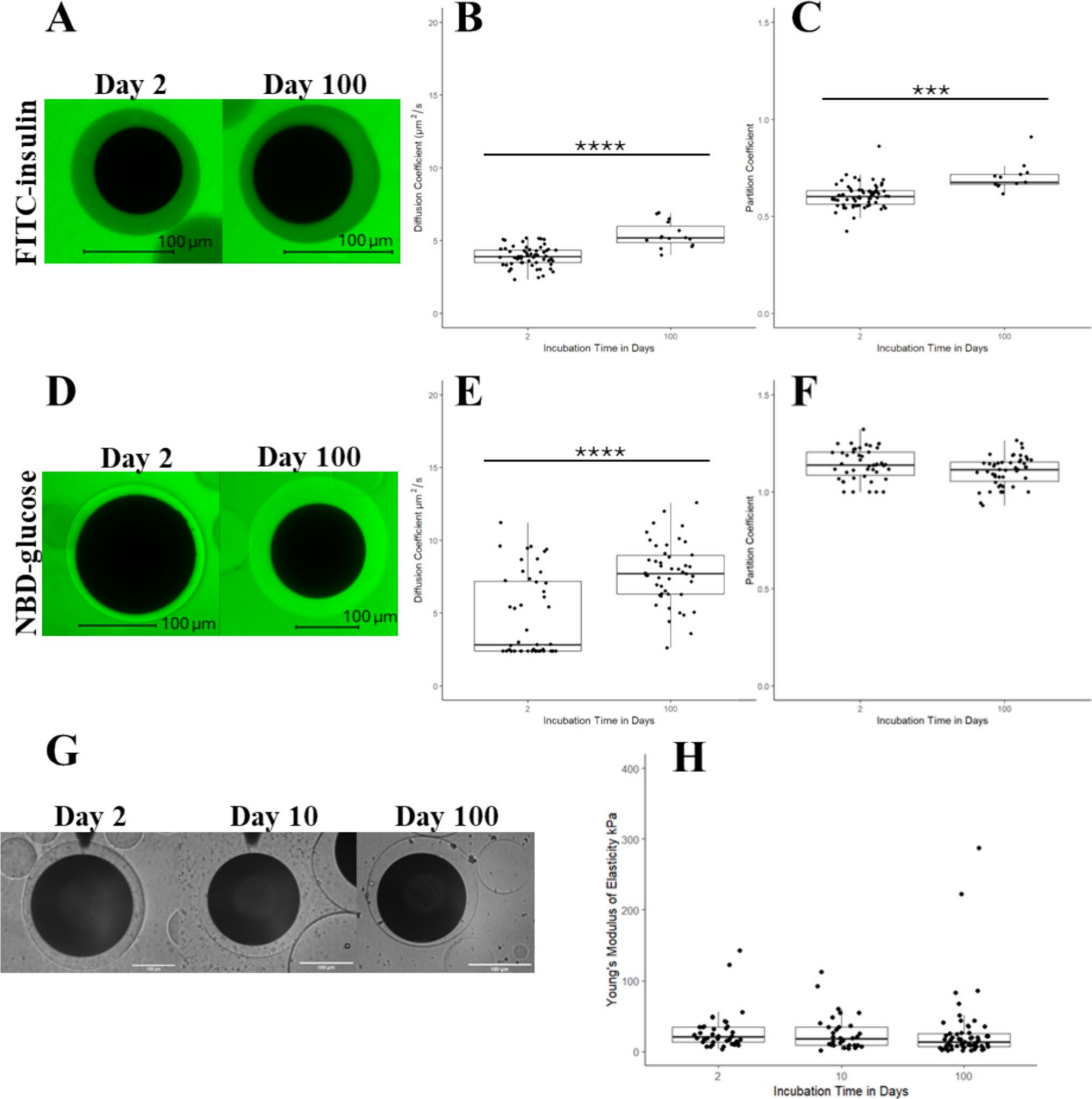
Characterization of the transport and biomechanical properties of conformally coated model beads over time. **A** Confocal images of conformal coated beads in FITC-insulin solution at day 2 and day 100 after fabrication. **B** Time comparison of FITC-insulin D in conformal coatings. **C** Time comparison of the FITC-insulin P at day 2 and day 100 after fabrication. **D** Confocal image of conformal coated bead in NBD-glucose solution at day 2 and day 100 after fabrication. **E** Time comparison of the NBD-glucose D. **F** Time comparison of the NBD-glucose P at day 2 and day 100 after fabrication. **G** Representative light microscope image of conformal coated islet model beads sampled by AFM on day 2, 10, and 100 after fabrication. (**H**) Day 2, day 10, and day 100 quantification of YM of conformal coated beads. Scale bars are 100µm. Single asterisk (p < 0.05), double asterisk (p < 0.01), triple asterisk (p < 0.001), quadruple asterisk (p < 0.0001)

FRAP was performed on individual conformally coated polystyrene islet model beads incubated overnight in the fluorescent analyte solution to measure D of the capsule hydrogel for different incubation times. Images from the confocal microscope can be seen in Fig. 3A and D, where the full dark circle is the bead and the ring around it is the conformal capsule. The average D for FITC-insulin through the conformal coating for the day 2 and day 100 time points (Fig. 3B) was 3.9 ± 0.7 µm^2^/s (range: 2.3-5.2 µm^2^/s) and 5.4 ± 0.9 µm^2^/s (range: 4.0-6.9 µm^2^/s), respectively. The means of these groups were found to be statistically significantly different from each other and showed an increase over a 100-day period.

The average NBD-glucose D values for the encapsulated beads for the day 2 and day 100 time points (Fig. 3E) were 4.7 ± 2.8 µm^2^/s (range: 2.4-11.2 µm^2^/s) and 7.7 ± 2.1 µm^2^/s (range: 2.6-12.6 µm^2^/s), respectively, higher than FITC-insulin D as expected given the smaller molecular size of NBD-glucose compared to FITC-insulin.

Confocal microscopy was used to determine P of individual conformal coated model beads in relation to the outer media. We found a statistical increase in average P for FITC-insulin over time, with day 2 and day 100 having partitionings of 0.61 ± 0.06 (range: 0.42-0.86) and 0.70 ± 0.07 (range: 0.61-0.91), respectively (Fig. 3C), showing an increase over a 100-day period.

For NBD-glucose, average P was 1.1 ± 0.08 (range: 1.0-1.3) and 1.1 ± 0.08 (range: 0.93-1.3) for the day 2 and day 100 groups, showing no significant change over time (Fig. 3F).

AFM was used to characterize YM of individual conformally coated polystyrene islet model beads incubated at different time points. Images from the AFM can be seen in (Fig. 3G), where the dark full circle is the bead and the transparent ring around it is the conformal capsule. No significant change in YM was found between the day 2, day 10, and day 100 groups, which had average values of 28.1 ± 27.6 kPa (range: 3.14-143.0 kPa), 25.8 ± 25.2 kPa (range: 1.81-112.4 kPa), and 27.0 ± 47.7 kPa (range: 1.41-288.0 kPa), respectively (Fig. 3H).

### Batch Variability

**Fig. 4:**
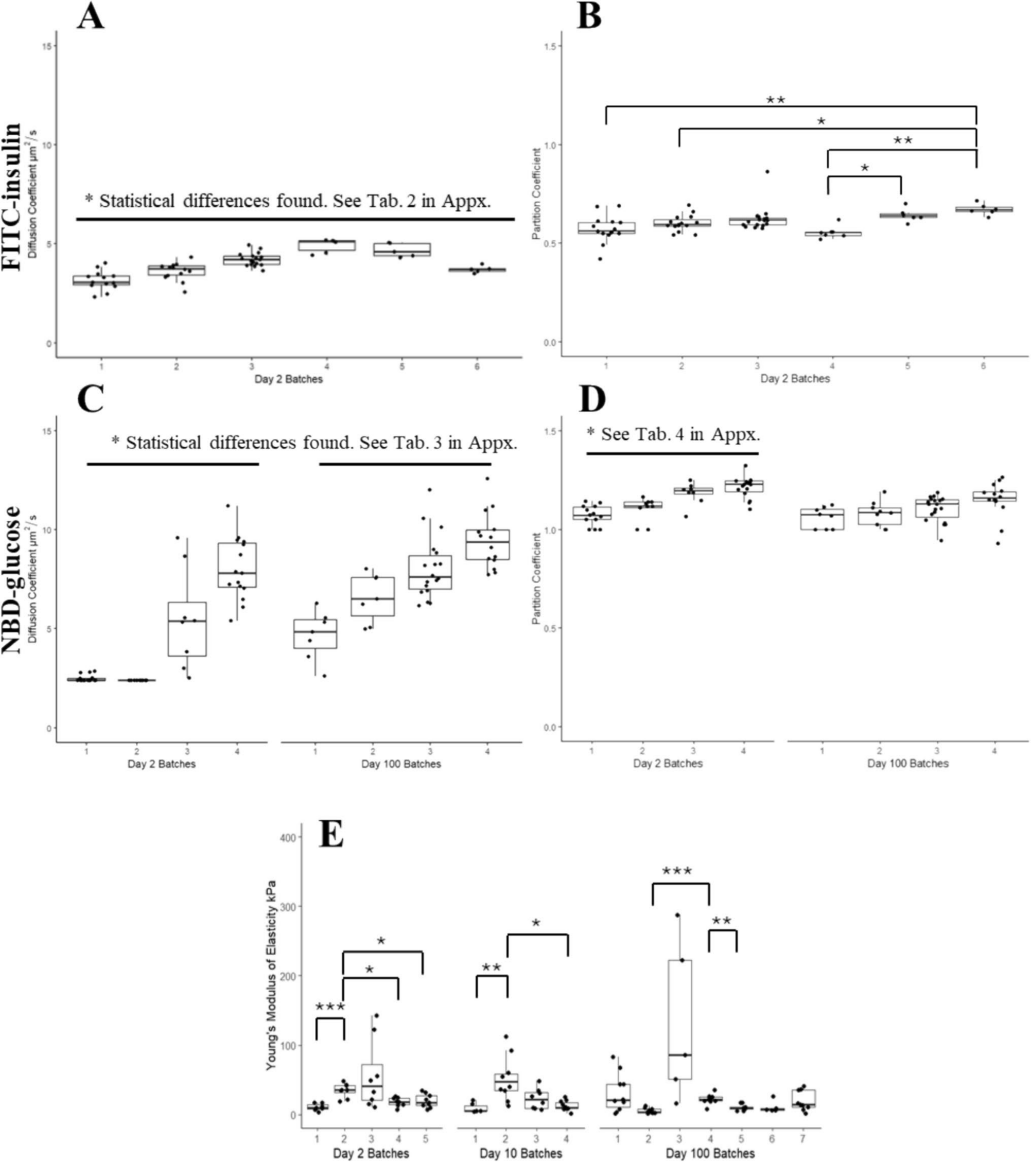
Characterization of the transport and biomechanical properties of different batches of conformally coated model beads. **A** Batch comparison of FITC-insulin D in conformal coatings. **B**. Batch comparison of the FITC-insulin P in conformal coatings. **C** Batch comparison of the NBD-glucose D at day 2 and day 100 after fabrication. **D** Batch comparison of the NBD-glucose P at day 2 and day 100 after fabrication. **E** Day 2, day 10, and day 100 batch quantification of YM of conformal coated beads. Single asterisk (p < 0.05), double asterisk (p < 0.01), triple asterisk (p < 0.001), quadruple asterisk (p < 0.0001)

Variability in D, P, and YM values from different batches were investigated. FRAP was performed and batch to batch variability was found in the measured values for both FITC-insulin and NBD-glucose D (Fig. 4A, C).

Confocal microscopy was used to determine P of individual conformal coated model beads in relation to the outer media. Batch variability was found in the measured values for both FITC-insulin and NBD-glucose P (Fig. 4B, D). However, the measured values for the day-100 NBD-glucose batches were consistent with each other.

The differences observed in means across batches may contribute to or explain the statistical differences observed at the day 2 and day 100 time points, suggesting that batch-level variability could be a confounding factor in the temporal analysis.

For the YM values measured using AFM, there was some variability between the batches (4-7) of each time point, with day 2 having the most inter batch-to-batch variability, batch 2 being statistically different from day 2 and day 10 batches, and day 100 having the largest range of values stemming within a single batch (batch 3) showing a wide range of 271.6kPa (Fig. 4E).

### Properties of coatings on Human Islets and Murine Nit1 PseudoIslets

**Fig. 5:**
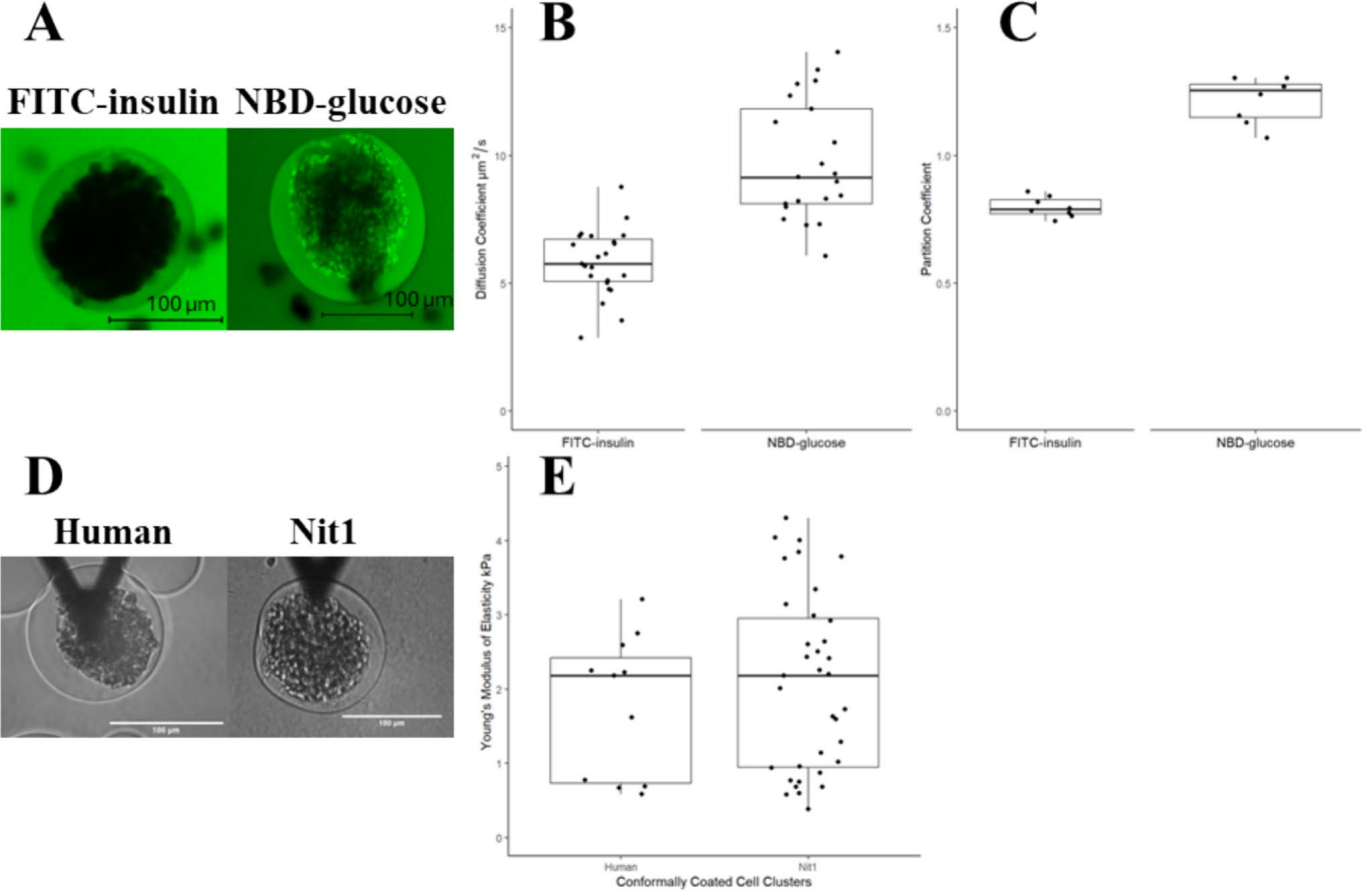
Characterization of the transport and biomechanical properties of different batches of conformally coated cell clusters. **A** Confocal image of conformal coated murine Nit1 pseudoislets in FITC-insulin and NBD-glucose. **B** Characterization of the diffusion coefficients (D) of conformally coated murine NIT-1 pseudoislets by FRAP. Comparison of the diffusion coefficient of FITC-insulin and NBD-glucose in conformal coatings. **C** Characterization of the partition coefficient (P) of conformally coated murine Nit1 pseudoislets. Comparison of the partition coefficient of FITC-insulin and NBD-glucose in conformal coatings. **D** Representative light microscope images of a conformal coated human islet and a conformal coated murine Nit1 pseudoislet. **E** Day 2 quantification of YM of conformal coated cell clusters. Scale bars are 100 µm.

The diffusion coefficients of individual conformally coated Nit1 pseudoislets were measured to have average values of 5.7 ± 1.2 µm^2^/s (range: 3.2-7.0 µm^2^/s) and 9.9 ± 1.9 µm^2^/s (range: 8.0-12.6 µm^2^/s) in FITC-insulin and NBD-glucose D, respectively (Fig. 5B). The partition coefficients for insulin and glucose of coated Nit1 pseudoislets were determined to be 0.8 ± 0.04 (range: 0.74-0.86) and 1.2 ± 0.09 (range: 1.1-1.3), respectively (Fig. 5C). AFM was used to characterize the YM of elasticity of individual coated primary human islets (Fig. 5E) and murine Nit1 pseudoislets (Fig. 5) incubated for 2 days. The coated human islets had an average YM value of 1.8 ± 0.95kPa (range: 0.59-3.21kPa) and the NIT-1 pseudoislets had an average value of 2.4 ± 1.4 kPa (range: 0.6-5.6 kPa) (Fig. 5). No statistical difference was found between the YM values of human islets and murine pseudoislets.

## Discussion

Through the conformal coating technique used in this study, individual model beads and primary and pseudo islets were encapsulated in PEG hydrogels. Atomic force microscopy (AFM) and fluorescence recovery after photobleaching (FRAP) were used to measure the mechanical and transport properties of these individually coated structures, a novel application of these techniques. These measurements provided valuable insights into the variability that may exist within the population of single encapsulated beads and cell clusters and across different time points of extended culture.

The conformal coating showed an increase in FITC-insulin and NBD-glucose diffusivity over a 100-day incubation period; however, it is important to note that these changes, although statistically significant (p < 0.05), were less than a factor of 2. Additionally, there was only one batch measured for the day 100 group for the FITC-insulin, which is a limitation of this study. FITC-insulin and NBD-glucose molecules were found to be able to move freely through the conformal coatings as indicated by the FITC-insulin and NBD-glucose P values. An increase in partitioning of FITC-insulin did occur over time. The conformal coating showed no change in NBD-glucose partitioning over a 100-day incubation period. Diffusivity and partitioning were higher for NBD-glucose compared to FITC-insulin, likely due to the difference in mass of the tagged molecules, as NBD-glucose has a molecular mass of 342.26 g/mol and FITC-insulin has a molecular mass of 5808 g/mol. However, it is important to note that other properties can affect diffusion, such as the polarity and solubility of the fluorescent molecules, as well as the presence of concentration gradients. The conformal coating maintained consistent YM values over a 100-day incubation period, which is an excellent indication that the polymeric coatings maintain their structural integrity over the 100-day time period.

The conformal coating procedure is consistently applied across all batches, with the only production-related differences being the date and time of encapsulation. To ensure reliability in characterizing the coating properties over time and between batches, we use model polystyrene beads for encapsulation. These beads are inert, even during long-term culture, unlike biological cells whose properties may vary depending on the batch and encapsulation timing. By coating model beads, we can confidently assess the performance and consistency of the conformal coating. This consistency allows us to directly compare batch data and monitor any trends or deviations in coating behavior.

Variability in diffusion coefficients were found between batches using statistical hypothesis testing. There was batch to batch variability in the day 2 batches measured in FITC-insulin as well as in the day 2 and 100 batches measured in glucose. Batch variability in P values was discovered for the FITC-insulin day 2 group and the glucose day 2 group. Statistical hypothesis testing revealed variability in YM between batches within each time point. Notably, the day 2 group exhibited the highest number of statistically significant differences between batches, while the day 100 group included only one batch (batch 3) with a particularly wide range of Young’s modulus values (271.6kPa). Aside from batch 3 on day 100, the YM values within each batch were closely distributed, suggesting that the production process exhibits minimal intra-batch variability. Average batch values and standard deviations can be found in the Appendix.

In total, FITC-insulin and NBD-glucose diffusivity values ranged from 2.3-6.9 µm^2^/s and 2.4-12.6 µm^2^/s, respectively, including temporal and batch variabilities FITC-insulin and NBD-glucose partitioning values ranged from 0.42-0.91 µm^2^/s and 0.93-1.3 µm^2^/s, respectively, including temporal and batch variabilities. YM values ranged from 1.41-288.0 kPa, including temporal and batch variabilities. The differences observed in means across batches may contribute as a confounding factor towards the statistical differences observed at the day 2 and day 100 time points. Although statistically significant differences were observed between batches, these differences in values are within the same order of magnitude and more investigation is needed to determine how biologically meaningful these differences between batches could be, and if these measured differences translate into different functionality of the coated product. Overall, while we found some statistical differences in the batch measurements of YM, D, and P for the conformally coated beads, these differences likely have minimal impact on the biological functions of conformal coated islets being the observed variations in the same range.

When comparing YM of the day 2 conformally coated cell clusters with the conformally coated polystyrene beads, we found that the conformally coated hydrogels on cell clusters were significantly less stiff than those on rigid polystyrene islet model beads. It is likely that the stiffness of the underlying cell clusters is affecting this measurement, compressing under the applied uniaxial stress of the AFM cantilever. Indeed, upon visual inspection of the AFM measurements using the light microscope, it could be seen that the individual cells would shift slightly with indentation, although the polystyrene bead would remain stable and immobilized as it acts as a rigid substrate.

The diffusion coefficients of coated cell clusters in both FITC-insulin and NBD-glucose were similar, although slightly higher to those of the day 2 coated beads, potentially due to the beta cells driving diffusion rates through the release or intake of insulin and glucose, respectively. The diffusion coefficients for FITC-insulin and NBD-glucose in PEG conformal coatings in this study were lower than in previously published work using bulk multi-arm PEG hydrogels, which gave a diffusivity range of 40-100 µm^2^/s [37]. However, this previously reported work quantified diffusion of a different analyte (dextran). Despite similar molecular weight (4kDa) to that of FITC-insulin (6kDa) and 2-NBD-glucose (3.4kDa), the noted discrepancy may be attributed to variations in hydrogel composition, and molecular conformations of dextran and insulin. Another study measured the rate of movement of NBD-glucose through human islets, determining that to be approximately 1.5 µm/min [38]. If we convert our lowest average diffusion value for NBD-glucose (Day 2, Batch 2) into average displacement over time using the root mean square (RMS) formula, we get approximately 16.9 µm/min, giving a much higher value.

The P values of the coated cell clusters were slightly higher than those of the day 2 coated beads, also likely due to the beta cells driving insulin and glucose diffusion and highlighting the importance of validating these methods using cell clusters. Additionally, D and P values were comparable for both coated beads and coated cell clusters with the same incubation period.

A key limitation of this study is that all experiments were conducted under *in vitro* conditions, which do not fully replicate the complexity and harshness of *in vivo* environments. Additionally, the immobilization of coated cell clusters for measurement may cause the cells to undergo significant hypoxia and potential structural change.

Of current micro-encapsulation technologies for islet transplantation, the longest *in vivo* viability of encapsulated human islets in rats is around 238 days. However, that study reported some device failure after 100 days [39]. Our approach enables investigating whether device failure could be due to capsule degradation over time with single capsule resolution. Previously reported approaches for characterization of encapsulation materials were limited to characterization of bulk coating materials rather than single devices. Our approach was able to determine that *in vitro*, PEG conformal coatings are stable, operational, and do not degrade for at least 100 days, a feature that is critical for long-term immunoisolation of coated therapeutic cells. In future studies, the same approach can be used to evaluate long-term time points, as well as more batches for the insulin day 100 time group, and samples retrieved after long-term implantation in diabetic models.

## Conclusion

In summary, we determined the transport and mechanical properties of this conformal coating over a 100-day time period. We also identified batch-to-batch variability in YM, as well as in D and P of the conformal coatings. Further investigation is warranted to determine the biological relevance of these variations, particularly in the context of pancreatic islet cell function. Importantly, we found that the conformal coatings remained functional over the 100-day time period, as demonstrated by the relatively stable mechanical properties. Thus, this conformal coating shows promising stability to enable long-term immunoisolation of encapsulated islets. Additionally, we demonstrated a novel measurement technique capable of assessing the mechanical and transport properties of individually coated samples, giving a more precise characterization of inherent variabilities within a sample population. Thus, this method could be employed for future batch validation studies and release criteria for clinical studies. While this report is focused on encapsulation of islets for T1D treatment, the findings are applicable to other therapeutic cell clusters and organoids for treatment of other conditions that could benefit from cell transplantation.

## Appendix

**Fig. 6:**
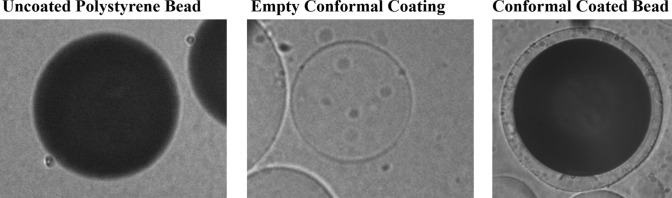
Images of model polystyrene beads before and after the conformal coating process.

See Fig. 6 and Tables 2, 3, 4, 5, 6, and 7.

**Table 2:** Adjusted p-values of compared batches for FITC-insulin diffusivity in conformal coated beads

Batches being compared (Batch No.)	Adjusted p-value
**2–1**	0.0245*
**3–1**	6.38E−9****
**4–1**	1.44E−11****
**5–1**	7.83E−9****
6–1	0.076
**3–2**	1.32E−3**
**4–2**	1.19E−7****
**5–2**	4.57E−5***
6–2	0.999
**4–3**	4.77E−3**
5–3	0.201
6–3	0.133
5–4	0.910
**6–4**	6.15E−5****
**6–5**	3.35E−3**

Single asterisk (p < 0.05), double asterisk (p < 0.01), triple asterisk (p < 0.001), quadruple asterisk (p < 0.0001)

**Table 3:** Adjusted p-values of compared batches for NBD-glucose diffusivity in conformally coated beads

Incubation time (days)	Batches being Compared (Batch No.)	Adjusted p-value
2	2-1	0.17
	**3-1**	0.048*
	**4-1**	7.93E−9****
	**3-2**	0.042*
	**4-2**	7.77E−9****
	4-3	0.609
100	2-1	0.057
	**3-1**	3.11E−4***
	**4-1**	9.49E−6****
	3-2	0.106
	**4-2**	0.002**
	4-3	0.061

Single asterisk (p < 0.05), double asterisk (p < 0.01), triple asterisk (p < 0.001), quadruple asterisk (p < 0.0001)

**Table 4:** Adjusted p-values of compared day-2 batches for NBD-glucose partition coefficients

Batches being Compared (Batch No.)	Adjusted p-value
2-1	0.558
**3-1**	0.002**
**4-1**	3.07E−8****
**3-2**	0.047*
**4-2**	0.002**
4-3	0.63

Single asterisk (p < 0.05), double asterisk (p < 0.01), triple asterisk (p < 0.001), quadruple asterisk (p < 0.0001)

**Table 5:** Diffusion coefficient (D) for each batch of conformally coated beads in each time group

Analyte	Incubation time (days)	Batch No.	Mean ± standard deviation (µm^2^/s)
Insulin	2	1	3.14 ± 0.47
		2	3.62 ± 0.44
		3	4.21 ± 0.34
		4	4.92 ± 0.35
		5	4.68 ± 0.36
		6	3.71 ± 0.19
	100	1	5.39 ± 0.88
Glucose	2	1	2.49 ± 0.17
		2	2.40 ± 0.00
		3	5.49 ± 2.53
		4	8.00 ± 1.57
	100	1	4.65 ± 1.24
		2	6.57 ± 1.23
		3	8.05 ± 1.58
		4	9.45 ± 1.42

**Table 6:** Partition coefficient (P) for each batch of conformally coated beads in each time group

Analyte	Incubation time (days)	Batch No.	Mean ± standard deviation
Insulin	2	1	0.57 ± 0.07
		2	0.60 ± 0.04
		3	0.62 ± 0.07
		4	0.55 ± 0.03
		5	0.64 ± 0.04
		6	0.67 ± 0.03
	100	1	0.70 ± 0.07
Glucose	2	1	1.07 ± 0.05
		2	1.10 ± 0.06
		3	1.18 ± 0.06
		4	1.21 ± 0.05
	100	1	1.07 ± 0.05
		2	1.09 ± 0.06
		3	1.10 ± 0.07
		4	1.15 ± 0.09

**Table 7:** Young's modulus (YM) of elasticity for each batch of conformally coated beads in each time group

Incubation time (days)	Batch No.	Mean ± standard deviation (kPa)
2	1	10.58 ± 5.02
	2	34.88 ± 10.14
	3	56.32 ± 49.96
	4	18.11 ± 6.60
	5	19.72 ± 9.68
10	1	9.13 ± 6.91
	2	51.56 ± 31.01
	3	22.61 ± 14.60
	4	12.60 ± 7.06
100	1	30.91 ± 27.66
	2	5.34 ± 3.66
	3	132.73 ± 116.75
	4	22.22 ± 6.82
	5	10.05 ± 4.50
	6	10.77 ± 8.80
	7	19.36 ± 14.22

## Declarations

### Data Availability

Further information and requests for resources and reagents should be directed to and will be fulfilled by the lead contacts, Alice Tomei (atomei@med.miami.edu) and Noel Ziebarth (nziebarth@miami.edu). All data reported in this paper will be shared by the lead contact upon request. This paper does not report original code. Any additional information required to reanalyze the data reported in this paper is available from the lead contact upon request.
